# Transit Time Theory for a Droplet Passing through a Slit in Pressure-Driven Low Reynolds Number Flows

**DOI:** 10.3390/mi14112040

**Published:** 2023-10-31

**Authors:** Spencer W. Borbas, Kevin Shen, Catherine Ji, Annie Viallat, Emmanuèle Helfer, Zhangli Peng

**Affiliations:** 1 Richard and Loan Hill Department of Biomedical Engineering, University of Illinois at Chicago, 1200 W Harrison St., Chicago, IL 60607, USA; sborba2@uic.edu; 2 Adlai E. Stevenson High School, 1 Stevenson Drive, Lincolnshire, IL 60069, USA; kevinshen2024@gmail.com; 3 New Trier High School, 385 Winnetka Avenue Winnetka, IL 60093, USA; 4 Aix Marseille Univ, CNRS, Centre Interdisciplinaire de Nanoscience de Marseille (CINAM), Turing Centre for Living Systems, 13009 Marseille, France

**Keywords:** microfluidics, creeping flow, two-phase flows, closed-form solutions

## Abstract

Soft objects squeezing through small apertures are crucial for many in vivo and in vitro processes. Red blood cell transit time through splenic inter-endothelial slits (IESs) plays a crucial role in blood filtration and disease progression, while droplet velocity through constrictions in microfluidic devices is important for effective manipulation and separation processes. As these transit phenomena are not well understood, we sought to establish analytical and numerical solutions of viscous droplet transit through a rectangular slit. This study extends from our former theory of a circular pore because a rectangular slit is more realistic in many physiological and engineering applications. Here, we derived the ordinary differential equations (ODEs) of a droplet passing through a slit by combining planar Poiseuille flow, the Young–Laplace equations, and modifying them to consider the lubrication layer between the droplet and the slit wall. Compared to the pore case, we used the Roscoe solution instead of the Sampson one to account for the flow entering and exiting a rectangular slit. When the surface tension and lubrication layer were negligible, we derived the closed-form solutions of transit time. When the surface tension and lubrication layer were finite, the ODEs were solved numerically to study the impact of various parameters on the transit time. With our solutions, we identified the impact of prescribed pressure drop, slit dimensions, and droplet parameters such as surface tension, viscosity, and volume on transit time. In addition, we also considered the effect of pressure drop and surface tension near critical values. For this study, critical surface tension for a given pressure drop describes the threshold droplet surface tension that prevents transit, and critical pressure for a given surface tension describes the threshold pressure drop that prevents transit. Our solutions demonstrate that there is a linear relationship between pressure and the reciprocal of the transit time (referred to as inverse transit time), as well as a linear relationship between viscosity and transit time. Additionally, when the droplet size increases with respect to the slit dimensions, there is a corresponding increase in transit time. Most notably, we emphasize the initial antagonistic effect of surface tension which resists droplet passage but at the same time decreases the lubrication layer, thus facilitating passage. Our results provide quantitative calculations for understanding cells passing through slit-like constrictions and designing droplet microfluidic experiments.

## 1. Introduction

Studies of droplets squeezing through small or narrow constrictions have been extensively performed for various applications, such as microfluidics or biomedical engineering [1]. Furthermore, extensive research has delved into the movement of biological cells navigating narrow passages within the human body, encompassing phenomena such as our previous modeling work on the filtration of red blood cells within the spleen [2], the process of white blood cell diapedesis [3], and the intricate journey of cancer cells during metastasis [4]. In our recent work, we conducted extensive microfluidic experiments and combined them with multiscale simulations to understand the critical conditions for red blood cells’ passage through splenic inter-endothelial slits (IESs) and the physical mechanisms that control their transit dynamics [2]. Although our computationally predicted transit time matches well with our experimental measurements, the simulations are expensive and less insightful than analytical theory. Additionally, in another recent work, we developed an analytical theory for a droplet passing through a circular pore [5], but the idealized circular pore is significantly different from the IES geometry we studied experimentally and computationally [2]. In this study, we extended our analytical theory of a circular pore to a slit for the transit time of a droplet.

In general cases under both in vivo and in vitro conditions, the speed at which droplets or cells pass through constrictions is a critical factor, yet its quantitative characterization remains unclear. For instance, red blood cells’ transient slowdown as they transit through splenic IESs can lead to capture and subsequent destruction by macrophages of the immune system. An in vitro example is that of droplets flowing through microfluidic devices, where droplet velocity is also important for effective manipulation and separation processes. Another instance where flow velocity is important is in the flow of ferrofluids into ducts of circular or rectangular cross-sections, where additional parameters like pressure drop, constriction cross-section size, and curvature can significantly impact the efficiency of heat exchangers and mixers [6].

Numerous experimental studies have been conducted to understand the dynamics of droplets, vesicles, and cells as they transit through constrictions. In 1967, Gregersen et al. investigated the passage of red blood cells through small pores created in paper [7]. The development of versatile microfluidic technology 20 years or so has vastly expanded the opportunities to explore the behavior of soft objects squeezing through constrictions of various size and geometry under controlled flows. Examples of microfluidic-based experiment are Ma et al., who studied the flow patterns within droplets navigating through rectangular microchannels [8], and Wang et al., who discussed the dynamic behavior of viscoelastic droplets in Y-shaped capillary channels [9]. Gambhire et al., followed by Moreau et al., examined red blood cells as they pass through submicron-wide slits designed to mimic the splenic IESs, highlighting the crucial role of red blood cells’ mechanical properties for efficient transit [2,10]. Ren et al. created a microfluidic device to study the biophysical characteristics and transit times of cells navigating cyclically through constrictions [11]. Finally, to relate transit time and object viscosity, Khan et al. studied cancer cells and viscous droplets flowing in confining microchannels [12]. Still, not all experiments are performed at the micrometer scale. For example, Chen et al. sought to discover the effects of droplet size (in the millimeter range), constriction dimensions, and applied pressure drop on the trajectory of droplets in irrigation and agricultural applications [13]. Nevertheless, despite all this extensive work, how the transit dynamics of these soft objects precisely relate to the setup parameters (applied pressure drop or constriction geometry) and object intrinsic properties remains unclear.

Apart from the above experimental approaches, the transit of soft objects through small apertures has been studied both from a theoretical and a computational point of view. First, theoretically, Zhang et al. developed analytical models for investigating the pressure and minimum impulse associated with a droplet’s passage through a circular pore under a constant flow rate [14,15,16]. However, in many applications, pressure control takes precedence over flow rate regulation. In addition, Jensen et al. employed an energy-based approach to examine the behavior of a bubble as it squeezes through a short constriction [17], while Marmur conducted an analysis of droplet penetration through a capillary under the influence of gravity [18]. In the case of vesicles, Gompper and Kroll developed an analytical solution to model the mobility of vesicles as they squeeze through a cylindrical pore past a threshold driving field strength [19]. When considering red blood cells, Waugh et al. formulated an analytical model to estimate transit times during egress in bone marrow [20]. When studying Stoke’s flow, Dassios and Vafeas developed a 3D concentric sphere model for particles in creeping flows and derived analytical expressions for the velocity, the total pressure, the angular velocity, and the stress tensor fields [21]. In addition, we recently developed an analytical study of droplet transit through a circular pore [5].

In addition to analytical models, a wide array of numerical methods has been harnessed to study the passage of droplets, vesicles, and cells through microfluidic pores, as reviewed by Zhang et al. in 2014 [22]. For instance, Barthes-Biesel made pioneering contributions by employing boundary integral simulations to investigate the passage of vesicles and capsules through constrictions in an axisymmetric configuration [23]. Similarly, Zinchenko and Davis applied a similar approach to explore the three-dimensional scenario of a droplet passing through constrictions formed between spheres [24].

Despite extensive prior research, understanding how the transit time of an object through a constriction changes with the constriction dimensions or the applied pressure drop and with the object intrinsic properties such as viscosity or surface tension remains unclear. Existing studies, such as those by Zhang et al. [14,15,16], often assume constant flow rates, while real-world applications frequently involve constant pressure drops. Additionally, microscale applications primarily exhibit creeping flows instead of finite Reynolds number flows [14,15,16]. Moreover, real-world constrictions often have rectangular cross sections rather than circular ones. In this study, we developed analytical models for droplets passing through slit-like constrictions under constant pressure conditions in order to derive the exact solution of transit time. Our work has two novelties: first, it is an analytical study, rather than a computational study, where it is rather trivial to change the constriction geometry; second, from the application perspective, the slit configuration is much more closely related to real in vivo situation like that of IESs, and our analytical approach gives a much more precise relationship between transit time and slit dimensions than a circular pore theory. Altogether, our results give much more insight into explaining the dynamics of cells passing through slits, which we previously observed in our experiments and simulations [2].

## 2. Theory of a Droplet Squeezing through an Infinitely Wide Slit

**Figure 1:**
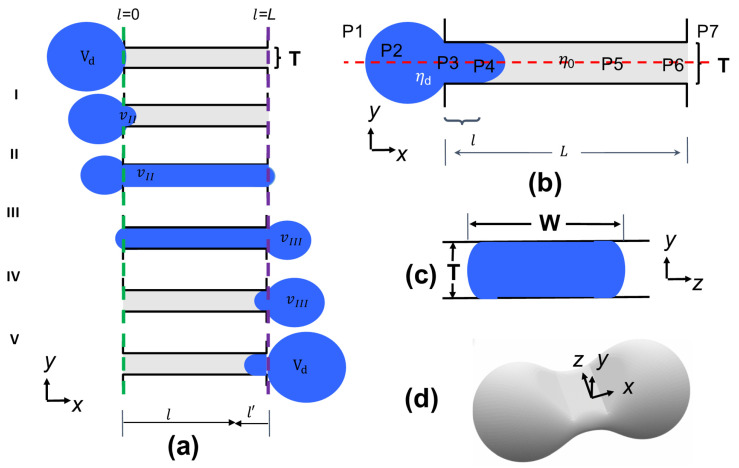
Sketch of the problem: a droplet of volume *V_d_* passes through a narrow slit of infinite width, thickness *T*, and length *L*. l is defined as the position along the length axis of the slit. $v_{II}$ is defined as the volume of the droplet that has passed the entrance of the slit (l=0) indicated by the green dashed line. $v_{III}$ is defined as the volume that has exited the slit (l=L) indicated by the purple dashed line. (**a**) Stages of the droplet passing through the slit. Stage I corresponds to the short process of developing a curved half-ellipsoidal shaped droplet head entering the slit. Stage II ends when $v_{II}=LWT+WT^{2}\pi /12$. Stage III ends when $v_{II}=V_{d}-WT^{2}\pi /12$. Stage IV ends when ${v_{III}=V}_{d}-WT^{2}\pi /12$. Stage V is the short process of the elliptical droplet head retracting to the radius of the spherical droplet. (**b**) Dimensions of the studied system (slit and droplet dimensions), viscosities of the droplet and external fluid, respectively, and pressures outside and along the droplet/slit (*P*1–*P*7). (**c**) Cross-section of the droplet in the slit. The slit is infinitely wide, but we assume that the droplet inside maintains a constant finite width *W*. (**d**) Three-dimensional view of the droplet in the slit.

Our model seeks to mathematically describe the forces acting on the droplet in each stage and at different locations. Figure 1a describes the position of the droplet at the boundaries of different stages and Figure 1b labels pressures *P*1 to *P*7 at different locations of the system during stage II. We take into account the lubrication layer between the droplet and the slit walls, and we consider the droplet and lubrication layer flows inside the slit to be planar Poiseuille flow and Couette flow, respectively. Forces due to surface tension are calculated at the spherical and/or elliptical ends of the droplet. The flow entering and exiting the slit is estimated with Roscoe’s solution with modifications to address the presence of multiple phases. The parameters that are used in the equations include the dimensions of an infinitely wide slit of thickness *T* and length *L*, the viscosity of the droplet *η_d_* and external fluid *η*_0_, the surface tension σ and volume $V_{d}$ of the droplet, and the pressure drop $\Delta P_{tot}=P1-P7$ that drives flow through the slit.

### 2.1. Contributions to Pressure Drop

We aimed to calculate the total transit time when the prescribed total pressure drop $∆P_{tot}$ is constant during the transit process. The total pressure drop $∆P_{tot}$ between the two sides along the dashed red path in Figure 1b can be grouped into four terms as:

(1) $$\Delta P_{tot}=P1-P7=∆P_{mem}+∆P_{poise,\eta_{d}}+∆P_{poise,\eta_{0}}+∆P_{roscoe}$$

where the first term $∆P_{mem}=P1-P2+P4-P5$ is the pressure term corresponding to pressure drops across the droplet membrane on both sides of the droplet. The second and third terms are the Poiseuille-related pressure drops, and the last term is the Roscoe flow term of a flow passing through an infinitely thin slit [25].

We assume that the width of the droplet inside the slit is constant, the same as the initial diameter of the droplet, given by $W=2{\left(3V_{d}/4\pi \right)}^{\frac{1}{3}}$. The flow rate *Q* due to pressure differential ∆*P_ηi_* and viscosity *η* inside the slit is represented by:

(2) $$Q=\frac{∆P_{\eta i}}{12\;L\eta }\;T^{3}W$$

which is the flow created between two infinitely long parallel plates, or planar Poiseuille flow. The equation can be rearranged to express the pressure drop due to viscous friction inside the slit:

(3) $$∆P_{poise}=\frac{12\;L\eta Q}{T^{3}W}$$

The pressure drop due to the viscous friction in the spherical part outside the slit can be found using Roscoe’s solution for the flow through a slit (Roscoe’s extension of Sampson flow [25]).

(4) $$∆P_{Roscoe}=\frac{32\;\eta Q\;}{\pi T^{2}W}$$

Thus, the total pressure drop of a droplet in an infinitely wide slit is represented as:

(5) $$∆P_{tot}=∆P_{mem}+∆P_{poise,\eta_{d}}+∆P_{poise,\eta_{0}}+∆P_{Roscoe}\;$$

### 2.2. Relationships between Pressure Drop and Lubrication Layer, Velocity Profile, and Flow Rate

**Figure 2:**
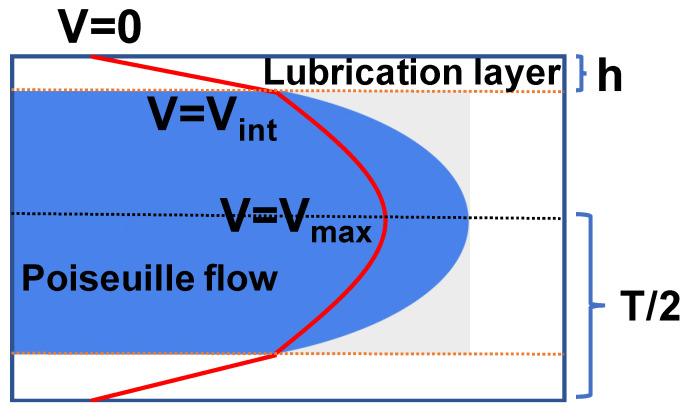
Velocity profile inside the slit with a lubrication layer between the droplet and the slit walls. The flow is a 2D Poiseuille flow inside the blue region.

As the droplet progresses through the slit, it does not touch the slit walls. Instead, the external fluid forms a lubrication layer between the droplet and each wall as shown in Figure 2, where *h* is the thickness of each layer. For a 2D droplet in a Hele–Shaw flow in a channel, it was shown that *h* is related to the channel height *T* and to the capillary number *Ca* as:

(6) $$h\left(V_{int}\right)=(2.1217/2)\;T\;{\left(Ca\right)}^{2/3}$$

Let $V_{int}$ be the velocity of the droplet at the droplet–lubrication layer interface and $V_{max}$ be the maximum velocity of the droplet. The shear stress is continuous across the interface, so that:

(7) $$\frac{\eta_{0}V_{int}}{h}=\frac{2\eta_{d}\left(V_{max}-V_{int}\right)}{r-h}$$

Note that this is exactly the same as the circular pore case (see Equation (7) in [5]).

To prepare to derive an analytical solution later, we define:

(8) $$\alpha =\frac{V_{max}-V_{int}}{V_{max}}$$

where α=1 for ideal Poiseuille flow and α=0 for ideal plug flow.

Let *Q*_1_ represent the flow rate of the droplet and *Q*_2_ represent the flow rate of a single lubrication layer. We calculate the flow rates as:

(9) $$Q=Q_{1}+{2Q}_{2}=\int_{h-r}^{r-h}V_{ave1}\;dA+2\int_{r-h}^{r}\frac{r-\rho }{h}V_{int}\;dA$$

Because in a planar Poiseuille flow, the average velocity is 2/3 of the maximum velocity, we have:

(10) $$V_{ave1}=2\left(V_{max}-V_{int}\right)/3+V_{int}=\frac{{2V}_{max}+V_{int}}{3}$$

(11) $$Q_{1}=\frac{2(r-h)\;\left({2V}_{max}+V_{int}\right)W}{3}$$

(12) $$Q_{2}=\int_{r-h}^{r}\frac{r-\rho }{h}V_{int}\;\;d\rho W=\frac{hV_{int}}{2}W\;$$

The total flow rate is:

(13) $$Q=Q_{1}+{2Q}_{2}=\frac{2(r-h)\;\left({2V}_{max}+V_{int}\right)W}{3}+hV_{int}W$$

This leads to our final equation, giving the total pressure drop $∆P_{tot}$ as function of the droplet velocities and flow rates:

(14) $$∆P_{tot}=2\sigma \left(1/R_{R}-1/R_{L}\;\right)+\frac{\left(V_{max}-V_{int}\right)\cdot 2\eta_{d}l}{{\left(r-h\right)}^{2}}+\frac{Q\cdot {3\eta }_{0}\left(L-l\right)}{{2r}^{3}W}+\frac{Q_{1}\cdot 8\frac{\eta_{d}}{2}}{{\pi \left(r-h\right)}^{2}W}+\frac{Q\cdot 8\frac{\eta_{0}}{2}}{\pi r^{2}W}\;$$

where $1/R_{R}$ and ${1/R}_{L}$ are the mean curvatures of the right and left ends of the droplet, respectively. Like the Sampson term shown in our previous work [5], the Roscoe term can be split into two terms of two half problems (the last two terms in Equation (14)). Equations (7) and (14) can be solved for $V_{int}$ and $V_{max}$.

### 2.3. Transit Times

Here, we determine the durations of the droplet transit stages and of its total transit through the slit, based on the pressure drops calculated above. To do so, we adapted the procedures used in the circular pore case [5]. For stages I and V, the time needed for the droplet to form or retract a curved half-ellipsoidal shaped droplet head is very small, and so they are considered negligible for this study.

Stage II:

During stage II, the constant total pressure ∆P_tot_ is:

(15) $$∆P_{tot}=2\sigma \left(1/R_{R}-1/R_{L}\;\right)+\frac{\left(V_{max}-V_{int}\right)\cdot 2\eta_{d}l}{{\left(r-h\right)}^{2}}+\frac{Q\cdot {3\eta }_{0}\left(L-l\right)}{{2r}^{3}W}+\frac{Q_{1}\cdot 8\frac{\eta_{d}}{2}}{{\pi \left(r-h\right)}^{2}W}+\frac{Q\cdot 8\frac{\eta_{0}}{2}}{\pi r^{2}W}$$

where $1/R_{R}=1/W+1/T$ when $R_{R}$ is approximated in a rectangular slit with dimensions W and T [26] and $R_{L}={\left[(V_{d}-lWT-T^{2}W\pi /12)/(4\pi /3)\right]}^{1/3}$. After solving Equation (15) with Equation (7) together, we obtain:

(16) $$\frac{dl}{dt}=V_{int}(l)$$

This nonlinear ODE can be solved by integrating from the initial condition $l\left(t=0\right)=0$ to $l\left(t_{2}\right)=L$. The unknown time $t_{2}$ then can be solved numerically.

Stage III:

The flow rate during stage III can be found using the pressure drop:

(17) $$∆P_{tot}=2\sigma \left(1/R_{R}-1/R_{L}\;\right)+\frac{\left(V_{max}-V_{int}\right)\cdot 2\eta_{d}L}{{\left(r-h\right)}^{2}}+\frac{Q_{1}\cdot 8\eta_{d}}{{\pi \left(r-h\right)}^{2}W}\;$$

where $R_{R}={\left[v_{III}/(4\pi /3)\right]}^{\frac{1}{3}}$ and $R_{L}={\left[V_{d}-LWT-v_{III})\right]}^{\frac{1}{3}}$, and $v_{III}$ is the volume of the droplet exiting the slit.

(18) $$Q\left(v\right)=\frac{dv_{III}}{dt}$$

The ODE of v(t) can be solved with initial condition $v_{III}\left(0\right)=2\pi \;(T^{2}W/8)/3=[T^{2}W\pi ]/12$. By numerically integrating from *t* = 0 to an unknown time $t_{3}$ where $v_{III}\left(t_{3}\right)=V_{d}-LWT-{[T}^{2}W\pi ]/12$, we can then numerically solve for $t_{3}$.

Stage IV:

Since stage II and stage IV are similar, $∆P_{tot}$ is found the same as in stage II except each appearance of l is replaced with l−L.

(19) $$∆P_{tot}=2\sigma \left(1/R_{R}-1/R_{L}\;\right)+\frac{\left(V_{max}-V_{int}\right)\cdot 2\eta_{d}(L-l)}{{\left(r-h\right)}^{2}}+\frac{Q\cdot {3\eta }_{0}l}{{2r}^{3}W}+\frac{Q_{1}\cdot 8\frac{\eta_{d}}{2}}{{\pi \left(r-h\right)}^{2}W}+\frac{Q\cdot 8\frac{\eta_{0}}{2}}{\pi r^{2}W}$$

where $R_{R}={\left[(V_{d}-(L-l)WT-T^{2}W\pi /12)/(4\pi /3)\right]}^{1/3}$ and $1/R_{L}=1/W+1/T$. If we denote $l=L-l^{\prime }$, we have:

(20) $$∆P_{tot}=2\sigma \left(1/R_{R}-1/R_{L}\;\right)+\frac{\left(V_{max}-V_{int}\right)\cdot 2\eta_{d}l^{\prime }}{{\left(r-h\right)}^{2}}+\frac{Q\cdot {3\eta }_{0}\left(L-l^{\prime }\right)}{{2r}^{3}W}+\frac{Q_{1}\cdot 8\frac{\eta_{d}}{2}}{{\pi \left(r-h\right)}^{2}W}+\frac{Q\cdot 8\frac{\eta_{0}}{2}}{\pi r^{2}W}\;$$

and

(21) $$\frac{dl^{\prime }}{dt}=V_{int}(l^{\prime })$$

with $R_{R}={\left[(V_{d}-(L-l)WT-T^{2}W\pi /12)/(4\pi /3)\right]}^{1/3}.$ This is a nonlinear ODE $l^{\prime }\left(t\right)$ which can be solved by integrating from the initial condition of $l^{\prime }\left(t=0\right)=0$ to $l^{\prime }{(t}_{4})=L$. This unknown time $t_{4}$ can then be solved numerically.

The transit time *t*_1_ and *t*_5_ of stages I and V are negligible as these are very short processes. Thus, the total transit time is:

(22) $$t_{T}=t_{2}+t_{3}+t_{4}$$

### 2.4. Relationship between Critical Pressure and Surface Tension

For given values of surface tension, droplet viscosity, and slit dimensions, the critical pressure ∆P_c_ is the minimum total pressure drop for which the droplet is able to transit completely through the slit, which is determined by the slit thickness *T*, the droplet volume $V_{d}$, width *W*, and its surface tension *σ* as:

(23) $$∆P_{c}\left(W,T,V_{d},\sigma \right)\approx 2\sigma \left\{\frac{1}{W}+\frac{1}{T}-{\left[{(V}_{d}-(W^{2}T\pi )/12)/(4\pi /3)\right]}^{-\frac{1}{3}}\right\}$$

For given values of pressure drop, droplet viscosity, and slit dimensions, the critical surface tension *σ*_c_ is the maximum surface tension for which the droplet is able to transit completely through the slit, which can be solved in Equation (23) by setting $∆P_{c}$ as the given pressure drop value.

### 2.5. Procedure to Obtain Numerical Solutions for Finite Surface Tension Cases

While an analytical solution can be found when the surface tension term $∆P_{mem}$ is absent, the differential equations in the above sections cannot be integrated manually when the surface tensions of both the right and left parts of the droplet are considered. In order to find the transit time when the surface tension is non-zero, we used the ODE solver ode45. In cases where ode45 failed to integrate an equation, the ode23 solver was used to perform the calculations needed to generate the figures in our study. We also applied the event function to calculate the transit times when certain conditions are met, such as $l\left(t_{2}\right)=L$ in Equation (16).

## 3. Results

With a mathematical explanation found to describe the system, analytical and numerical approaches were used to find transit times under various conditions. Emphasis was placed on the effects of adjusting individual parameters and on relationships that were found to be linearly related.

### 3.1. Analytical Solution for a Slit without Surface Tension but Constant α

In the specific condition where the surface tension is zero, we can derive the analytical solution of the transit times. We begin by analyzing the transit time equation for stage III, because the equations for stage II and IV are similar and will be discussed together immediately after stage III. α describes the extent to which droplet flow follows Poiseuille flow (α=1) or plug flow (α=0).

Stage III:

The volume of the droplet exiting the slit is:

(24) $$v=\frac{∆P_{tot}\cdot t\;}{\;\alpha \cdot \frac{12\;L\eta_{d}}{T^{3}W}+\frac{32\;\eta_{d}\;}{\pi T^{2}W}}+v_{0}$$

With initial conditions $v(0)=v_{0}=[T^{2}W\pi ]/12$, and $v\left(t_{3}\right)=V_{d}-LWT-[T^{2}W\pi ]/12$, at the end of stage III, we obtain:

(25) $$\frac{∆P_{tot}\cdot t_{3}\;}{\alpha \cdot \frac{12\;L\eta_{d}}{T^{3}W}+\frac{32\eta_{d}\;}{\pi T^{2}W}}+[T^{2}W\pi ]/12=V_{d}-LWT-[T^{2}W\pi ]/12$$

Thus

(26) $$∆P_{tot}\cdot t_{3}=\left(\alpha \cdot \frac{12\;L\eta_{d}}{T^{3}W}+\frac{32\eta_{d}\;}{\pi T^{2}W}\right)(V_{d}-LWT-[T^{2}W\pi ]/6)$$

Finally, $t_{3}$ is found to be:

(27) $$t_{3}=\frac{\left(\alpha \cdot \frac{12\;L\eta_{d}}{T^{3}W}+\frac{32\;\eta_{d}\;}{\pi T^{2}W}\right)\left(V_{d}-LWT-\frac{T^{2}W\pi }{6}\right)}{∆P_{tot}}$$

If $V_{d}=4/3\cdot \pi \;R^{3}>>LWT+\frac{T^{2}W\pi }{6}$, i.e., the droplet volume is much larger than the slit volume:

(28) $$t_{3}=\frac{\left(\alpha \cdot \frac{12\;R^{3}L\eta_{d}}{T^{3}W}+\frac{32R^{3}\;\eta_{d}\;}{\pi T^{2}W}\right)\left(4/3\cdot \pi \right)}{∆P_{tot}}\;$$

Stages II and IV:

As stated earlier, stages II and IV are similar; thus, the velocity of the droplet front (stage II) or rear (stage IV) in the slit is:

(29) $$U\left(l\right)=\frac{dl}{dt}=\frac{∆P_{tot}}{\;\frac{12L\;\eta_{0}}{T^{2}}+\frac{12l\left(\alpha \cdot \eta_{d}-\eta_{0}\right)}{T^{2}}+\frac{32\;\frac{{(\eta }_{d}+\eta_{0})}{2}\;}{\pi T}}$$

Assuming that $a=\frac{12(\alpha \cdot \eta_{d}-\eta_{0})}{T^{2}}$ and $b=\frac{12L\;\eta_{0}}{T^{2}}+\frac{32\;\frac{{(\eta }_{d}+\eta_{0})}{2}\;}{\pi T}$, we obtain:

(30) $$\frac{dl}{dt}=\frac{∆P_{tot}}{a\cdot l+b}$$

Using the separation of variables as before we find that:

(31) $$\int (a\cdot l+b)dl=\int ∆P_{tot}\;dt$$

(32) $$\frac{1}{2}al^{2}+b\cdot l=∆P_{tot}\cdot t+c$$

and that the limit conditions are:

l=0 at t=0          c=0

$$l=L\;at\;t_{2//4}\;\;\;\;\frac{1}{2}aL^{2}+b\cdot L=∆P_{tot}\cdot t_{2//4}$$

We obtain:

(33) $$t_{2//4}=\frac{\frac{1}{2}aL^{2}+bL}{∆P_{tot}}\;$$

(34) $$t_{2//4}=\frac{\frac{6\left(\alpha \cdot \eta_{d}-\eta_{0}\right)}{T^{2}}L^{2}+\left(\frac{12L\;\eta_{0}}{T^{2}}+\frac{32\;\frac{{(\eta }_{d}+\eta_{0})}{2}\;}{\pi T}\right)L}{∆P_{tot}}$$

To conclude, for the case of zero surface tension, σ=0, the total transit time is:

(35) $$t_{T}≅t_{2}+t_{3}{+t}_{4}$$

(36) $$t_{T}=t_{2}+t_{3}{+t}_{4}=\frac{\frac{12\left(\alpha \cdot \eta_{d}-\eta_{0}\right)}{T^{2}}L^{2}+2\left(\frac{12L\;\eta_{0}}{T^{2}}+\frac{32\;\frac{{(\eta }_{d}+\eta_{0})}{2}\;}{\pi T}\right)L}{∆P_{tot}}+\frac{\left(\alpha \cdot \frac{12\;R^{3}L\eta_{d}}{T^{3}W}+\frac{32R^{3}\;\eta_{d}\;}{\pi T^{2}W}\right)\left(4/3\cdot \pi \right)}{∆P_{tot}}\;$$

In the case of an ideal plug flow (α=0) and ${if\;t}_{3}>>t_{2//4}$ and $V_{d}>>LWT$

(37) $$\frac{\eta_{d}}{∆P_{tot}\;t}=\frac{T^{2}W}{32R^{3}\left(4/3\right)}$$

### 3.2. Effects of Pressure Drop, Slit Dimensions, and Droplet Properties on Transit Times

We next analyzed the dependence of the total transit time on the various parameters of our system and dissected the transit time from different stages. To analyze the transit time of a droplet squeezing through a slit, we studied the effect of the total pressure drop $∆P_{tot}$, slit dimensions (length L and thickness *T*), and droplet parameters (surface tension σ, volume $V_{d}$, and viscosity $\eta_{d}$). We investigated the effects of each of these variables on the total transit time t_T_ or on the reciprocal of the transit time 1/*t*_T_ (referred to as inverse transit time). We also investigated the effects of these variables on the transit and inverse transit time of each stage (*t*_2_, *t*_3_, *t*_4_ and 1/*t*_2_, 1/*t*_3_, 1/*t*_4_). In our study, we used a standard case with a pressure drop of 300 Pa, a slit length of 4 µm, a droplet volume of 73.6 µm^3^, a droplet viscosity of 0.01 Pa s, and a solution viscosity of 0.0012 Pa s. These values for droplet properties were obtained from a typical red blood cell [2]. In addition, we used a slit thickness of 0.5 µm and a droplet width of 5 µm inside the slit. We studied the surface tension in the order of 20 pN/µm, in the range of the membrane surface tension of red blood cells passing through splenic slits estimated in our recent work [2]. Using these values as a baseline, the effect of each variable on transit time was investigated.

We investigated the effects of the aforementioned variables by writing functions in Matlab, using the equations for the total transit time and stage transit times that we derived above. These Matlab functions calculate the transit time when given values for the studied parameters. To obtain the relationships between transit time and various parameters, we isolated the individual parameters and calculated transit time across a reasonable range for that isolated parameter.

#### 3.2.1. Effects of Pressure Drop and Droplet Viscosity

**Figure 3:**
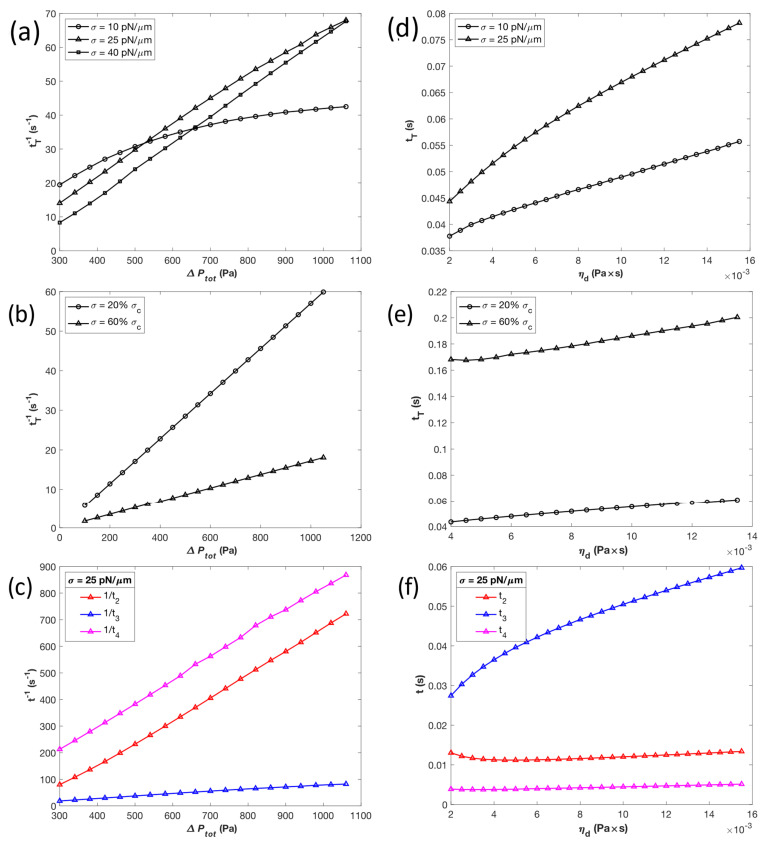
Effects of pressure drop $∆P_{tot}$ and droplet viscosity *η_d_* on transit times with finite values of surface tension *σ*. (**a**) Effect of $∆P_{tot}$ with constant values of *σ* on the inverse of the total transit time 1/*t*_T_. (**b**) Effect of $∆P_{tot}$ with *σ* as a fixed percentage of the critical surface tension value *σ*_c_ on 1/*t*_T_. (**c**) Effect of $∆P_{tot}$ on inverse transit times of stages II to IV, 1/*t*_i_, for *σ* = 25 pN/µm. (**d**) Effect of *η_d_* with constant *σ* values on transit time *t*_T_. (**e**) Effect of *η_d_* with *σ* as fixed percentage of *σ*_c_. (**f**) Effect of *η_d_* on transit times of stages II to IV, *t*_i_, for *σ* = 25 pN/µm.

For constant surface tensions *σ* of 10, 25, and 40 pN/µm, the inverse transit time 1/*t*_T_ was obtained for pressure drops $∆P_{tot}$ from 1100 down to 300 Pa, which is the physiological range of pressure drop in the spleen for RBC filtration [2], as shown in Figure 3a. The inverse time vs. pressure drop (1/*t*_T_ vs. $∆P_{tot}$) curves for surface tensions of 25 and 40 pN/µm are approximately linear for all pressure drops, but for *σ* = 10 pN/µm the 1/*t*_T_ vs. $∆P_{tot}$ curve is approximately linear only above 650 Pa. Below that pressure drop, there appears to be a downwards curvature until the critical pressure is reached. The dependence of 1/*t*_T_ with increasing $∆P_{tot}$ also changes with *σ*. For example, the 10 pN/µm droplet has the highest relative inverse transit time at lower pressure drops, before dropping to having the lowest inverse transit time compared to the two other values of σ after $∆P_{tot}$ increases beyond 650 Pa. Conceptually, surface tension resists the droplet’s progression through stage II, but a decreased capillary number is associated with a thinned lubrication layer. It is possible that a surface tension of 10 pN/µm at low pressure drop means that the droplet does not resist entry into the slit and is slow enough to not have a high capillary number, but increasing the pressure drop increases velocity, and therefore capillary number and lubrication layer thickness. Regardless, inverse transit time is shown to increase with pressure drop.

We also investigated the dependence of the inverse transit time 1/*t*_T_ on total pressure drop $∆P_{tot}$ when the droplet surface tension σ is not constant but is a percentage of the critical surface tension *σ*_c_. When given values of pressure drop, droplet viscosity, and slit dimensions, the critical surface tension *σ*_c_ is the maximum surface tension for which the droplet is able to transit completely through the slit. If *σ* ≥ *σ*_c_, then the drop will not pass through the slit. The 1/*t*_T_ vs.$\;\eta_{d}$ curves were obtained for surface tensions set to 20% and 60% *σ*_c_ (Figure 3b). A higher percentage of *σ*_c_ resulted in a longer transit time compared to the lower percentage. However, in both cases, there was a strong linear relationship between $∆P_{tot}$ and 1/*t*_T_ for all pressure drops in the range studied. An 11-fold increase in $∆P_{tot}$, from 100 Pa to 1100 Pa, corresponded to an approximately 11-fold increase in 1/*t*_T_ for both situations, with 1/*t*_T_ going from 5.71 s^−1^ to 62.73 s^−1^ at 20% *σ*_c_, and from 1.72 s^−1^ to 18.93 s^−1^ at 60% *σ*_c_. While both cases start with similar 1/*t*_T_ values at low $∆P_{tot}$, 1/*t*_T_ increases at a higher rate for 20% *σ*_c_, with a slope of about 0.057 Pa^−1^ s^−1^, compared to a slope of about 0.017 Pa^−1^ s^−1^ at 60% *σ*_c_.

The effects of total pressure drop $∆P_{tot}$ on the inverse transit times 1/*t*_i_ for stages II, III, and IV at a surface tension of 25 pN/µm can be observed in Figure 3c. For all stages, their inverse transit time appears to have a linear relationship with $∆P_{tot}$ for all pressure drops in the range studied. Stage III is the dominant phase of the droplet passing through a slit when the surface tension is not near critical, with the lowest inverse transit time (and thus the highest transit time), while stage IV has the lowest relative transit time.

For droplet viscosities *η_d_* ranging from around 0.002 to 0.015 Pa s, transit time *t*_T_ was obtained at constant surface tensions *σ* of 10 and 25 pN/µm (Figure 3d) and at 20% and 60% *σ*_c_ (Figure 3e). The transit times for droplets with surface tensions of 10 and 25 pN/µm appear to converge at lower *η_d_*, reaching values of 37.8 and 44.3 ms at 0.002 Pa s, respectively (Figure 3d). However, as *η_d_* increases, the transit time of the droplet with 10 pN/µm surface tension increases at a faster rate than the one at 25 pN/µm. There also appears to be a linear relationship in both cases when *η_d_* increases beyond 0.006 Pa s. Similar results were observed when *t*_T_ was plotted against *η_d_* at 20% and 60% σ_c_ (Figure 3e). Both Figure 3d,e were produced at the standard total pressure drop of 300 Pa. Figure 3f illustrates the relationship between *η_d_* and transit times *t*_i_ for stages II, III, and IV at *σ* = 25 pN/µm. Once again, the total transit time was most greatly influenced by stage III, while stages II and IV made a significantly smaller contribution, with negligible change in their transit times as viscosity was increased.

#### 3.2.2. Effects of Slit and Droplet Dimensions

**Figure 4:**
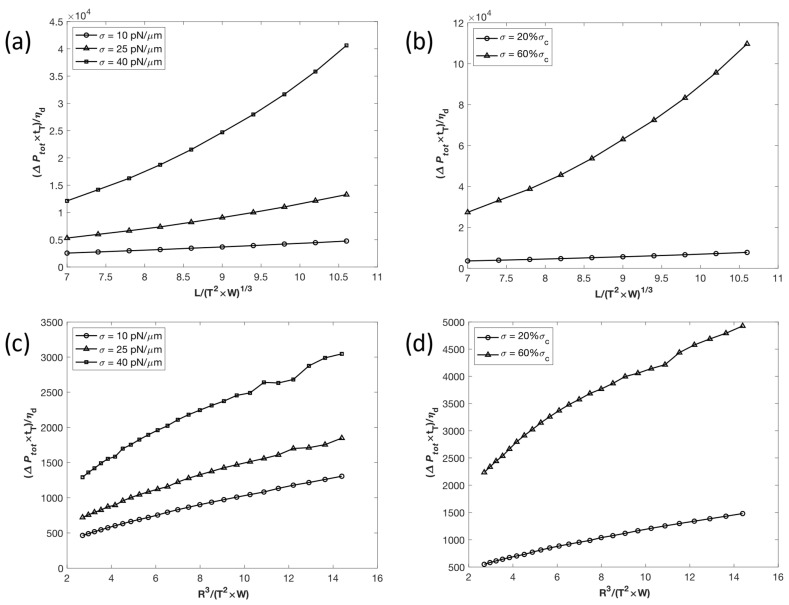
Effects of slit dimensions (ratio of length *L* and size (*T*^2^ × *W*)^1/3^) on transit time *t*_T_ with finite values of surface tension *σ*. (**a**) Effect of slit dimension ratio with constant *σ* values. (**b**) Effect of slit dimension ratio with *σ* as a fixed percentage of critical surface tension *σ*_c_. (**c**) Effect of slit width *W* and thickness *T* with constant σ values. (**d**) Effect of *W* and *T* with *σ* as a fixed percentage of *σ*_c_ value.

We utilized the expression (*T*^2^ × *W*)^1/3^ to represent the size of the rectangular slit. The relationship between transit time *t*_T_, in the form of the dimensionless variable ($∆P_{tot}$*t*_T_)/*η_d_*, and the ratio between the slit length *L* and size was obtained for (*T*^2^ × *W*)^1/3^ values from 7 to 10.5, both at constant surface tensions *σ* and at percentages of *σ*_c_ (Figure 4). When comparing ($∆P_{tot}$t_T_)/*η_d_* to (*T*^2^ × *W*)^1/3^ at constant *σ*, as shown in Figure 4a, there is an approximately linear relationship, potentially with a slight upward concavity. For all assessed values of (*T*^2^ × *W*)^1/3^, *t*_T_ is higher for *σ* = 25 pN/µm than for *σ* = 10 pN/µm. Additionally, *t*_T_ increases at a significantly higher rate when the surface tension is 25 pN/µm.

In Figure 4b, we see the effect of *L*/(*T*^2^ × *W*)^1/3^ on ($∆P_{tot}$*t*_T_)/*η_d_* with surface tensions set at 20% and 60% *σ*_c_. Similar to conditions at constant σ, there is an approximately linear relationship, with ($∆P_{tot}$*t*_T_)/*η_d_* increasing at a higher rate when the surface tension is at a higher percentage of *σ*_c_.

The effect of slit size on transit time *t*_T_ with constant surface tension *σ* values is shown in Figure 4c, plotted in the dimensionless variables ($∆P_{tot}$*t*_T_)/*η_d_* vs. *R*^3^/(*T*^2^ × *W*), with *R*^3^/(*T*^2^ × *W*) values ranging from 2.70 to 15.17. At lower *σ* values of 10 and 25 pN/µm, the droplet appears to behave similarly in terms of transit time, with *t*_T_ increasing at a slightly faster rate at *σ* = 25 pN/µm and both having an approximately linear relationship. At *σ* = 40 pN/µm, the droplet behavior is similar to that calculated at lower *σ* cases up to *R*^3^/(*T*^2^ × *W*) = 10, but as the droplet volume increases further, the ($∆P_{tot}$*t*_T_)/*η_d_* dependence on *R*^3^/(*T*^2^ × *W*) becomes more nonlinear and displays irregularities.

When the effect of slit size on transit time was plotted with *σ* set at a fixed percentage of the critical surface tension *σ*_c_, an approximately linear relationship with a slight downwards concavity was observed, as seen in Figure 4d. When *σ* = 60% *σ*_c_, *t*_T_ increases at a slightly higher rate, with a slope of around 227, compared to a slope of around 74 when *σ* = 20% *σ*_c_.

#### 3.2.3. Effect of Surface Tension

**Figure 5:**
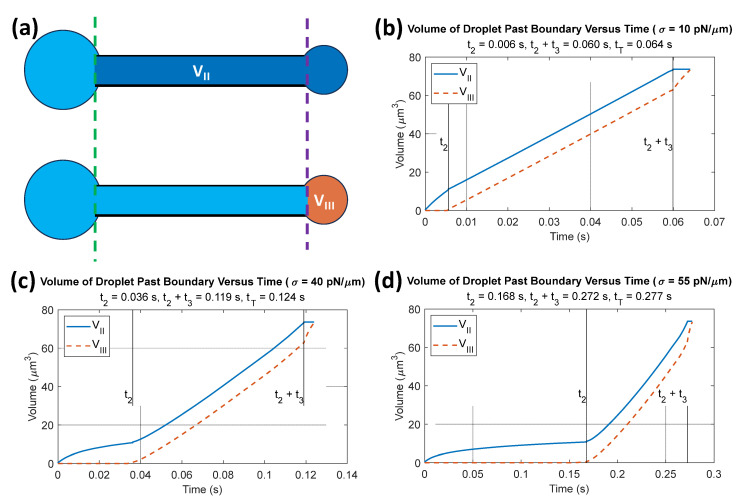
Volumes of the droplet that have passed the left and right openings of the slit (**a**) over time for droplet surface tension *σ* values of (**b**) 10, (**c**) 40, (**d**) and 55 pN/µm. Vertical lines delineate the time boundaries between stages II, III, and IV. Volume $v_{II}$ remains constant at stage IV due to full crossing of the slit’s left opening, and volume $v_{III}$ remains constant during stage II since the droplet does not cross the slit’s right opening until stage III.

Surface tension *σ* in the droplet serves as a source of nonlinearity in the progression of the droplet’s volume through the slit. Visualization of the progress of each stage at a pressure drop $∆P_{tot}$ of 300 Pa for various *σ* can be seen in Figure 5. The volumes of the droplet that have crossed the slit entrance and exit defined in Figure 1a are schematized in Figure 5a: $v_{II}$ represents the volume of the droplet that has passed the left opening, i.e., the entrance (dashed green line), and $v_{III}$ represents the volume of the droplet that has passed the right boundary, i.e., the exit (purple dashed line). The time evolution of $v_{II}$ and $v_{III}$ is shown in Figure 5b–d for surface tensions of 10, 40, and 55 pN/µm. Vertical lines delineate the time boundaries between stages II, III, and IV at *t*_2_ and *t*_2_ + *t*_3_ in Figure 5b–d. Note that when the volume remains constant, such as for $v_{II}$ during stage IV and $v_{III}$ during stage II, this indicates that the droplet has not yet or has already crossed the corresponding slit boundary. In Figure 5b, a surface tension of 10 pN/µm leads to the majority of the transit time being spent in stage III, as this stage involves the largest droplet volume transfer. As σ is increased from 10 to 55 pN/µm (Figure 5b–d), all stage transit times increase, but stage II transit time *t*_2_ increases and eventually exceeds stage III transit time *t*_3_. Conceptually, the net force due to the droplet surface tension opposes that of the droplet flow in the slit during all of stage II, but only half of stage III. As σ increases, this effect becomes more pronounced, and the volume flow rate becomes more nonlinear since the droplet curvature changes nonlinearly. With regards to the concavity of $v_{II}$ in stage II, surface tension on one hand would cause positive concavity due to decreasing curvature of the left droplet head. As volume leaves the left droplet head and enters the slit, its curvature gradually decreases and becomes more similar to that of the right droplet head, reducing the surface tension force that resists the droplet’s flow. On the other hand, a higher capillary number means thicker lubrication layers that resist droplet flow. In addition, as the droplet enters the slit, the average viscosity in the slit increases which means more resistance to flow. Figure 5c,d show visible negative concavity of $v_{II}$ in stage II, indicating that even though surface tension would otherwise cause positive concavity, lubrication layer thickness and viscous effects serve as negative feedback to droplet flow.

**Figure 6:**
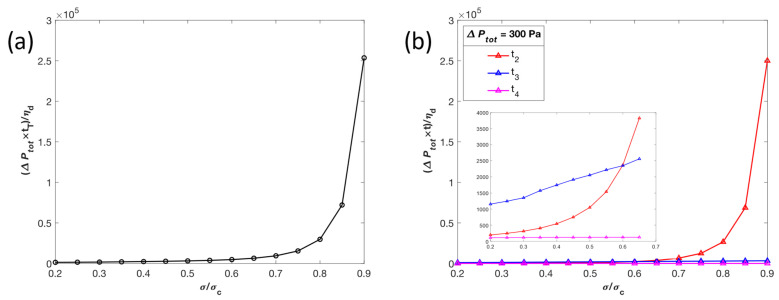
Effects of the surface tension *σ* as a percentage of critical surface tension *σ*_c_ on total transit time *t*_T_ (**a**) and stage transit times *t*_i_ (**b**) under a total pressure drop $∆P_{tot}$ of 300 Pa. The transit times have been normalized as ($∆P_{tot}$ *t*)/*η_d_*. This normalization process allows us to isolate the effect of increasing surface tension.

The effect of surface tension *σ* as an increasing percentage of critical surface tension *σ*_c_, ranging from 20% to 90% *σ*_c_, on total transit time *t*_T_ is shown in Figure 6a. The transit time increases exponentially as *σ* approaches *σ*_c_ and it can be assumed that the droplet will not pass through the slit when *σ* reaches the critical value. Figure 6b shows in more detail how the stage II, III, and IV transit times, *t*_2_ to *t*_4_, contribute to *t*_T_ across the same 20–90% range of σ_c_. As σ increases as a percentage of σ_c_, the stage IV transit time does not change significantly, stage III transit time increases approximately linearly, and the stage II transit time increases exponentially and exceeds *t*_3_ at σ beyond 60% *σ*_c_. As a result, between 20% and 60% *σ*_c_, stage III controls most of the total transit time behavior, while above 60% *σ*_c_, stage II drives the exponential increase in the total transit time.

The non-monotonic behavior observed in Figure 6 can be explained as follows. The surface tension has two effects on the transit time. Firstly, in stage III (Equation (17)), the surface tension term (the first term on the right-hand side of Equation (17)) resists the passage of the droplet due to the size difference between the two spheres on the two ends of the droplet in the first half of stage III and facilitates the passage in the second half of stage III. On the other hand, *σ* also changes the lubrication layer thickness *h* (Equation (6)). For the first half of stage III until the two spheres become equal, increased σ increases the resistance, therefore increasing the transit time. But the increased *σ* also decreases *h* (Equation (6)). With a decreased *h*, the flow rate increases, therefore decreasing the transit time in the second half of stage III. These two effects of surface tension can create a non-monotonic dependence of transit time on surface tension.

### 3.3. Comparison between the Current Slit Model and the Previous Circular Pore Model

**Figure 7:**
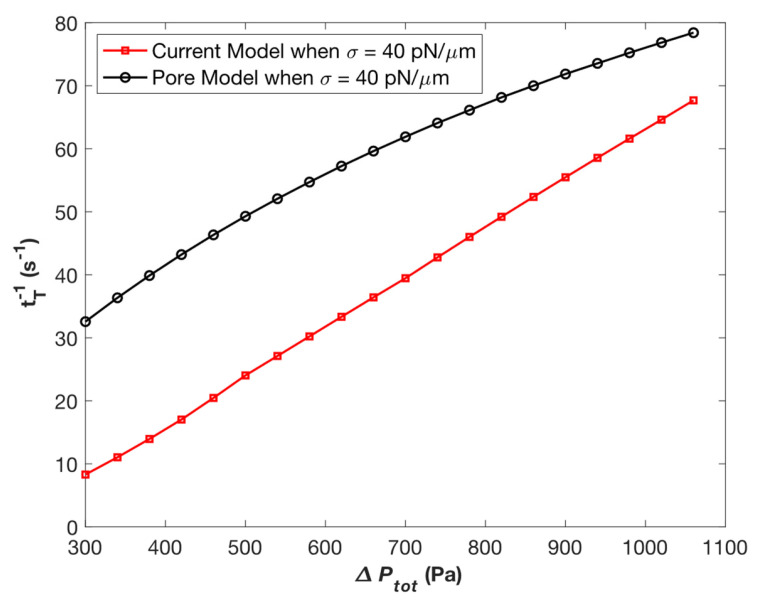
Effects of pressure drop $∆P_{tot}$ on inverse transit time 1/*t*_T_ when *σ* = 40 pN/µm for both the current slit model as well as the previous circular pore model.

There does not exist any other published paper that derives analytical equations for droplet transit time through rectangular slits to compare our results with. However, we conducted a comparison with the model developed by Tang et al. which analyzes droplet motion through a pore with a circular cross-section [5]. We conducted this comparison by utilizing a rectangular slit constriction as well as a circular pore constriction with the same cross-sectional area of 2.5 pm^2^, while keeping all other parameters such as surface tension and viscosity constant (Figure 7). We believe that the differences in the observed relationship are because of the changed constriction geometry.

## 4. Conclusions and Discussion

This paper presents analytical approaches to understand the passage of a droplet through a narrow slit. These approaches can have many applications in fields ranging from understanding the flow of biofuel and ethanol droplets [27] to improving the properties of agricultural pesticides in emulsion form [28]. Additionally, this model of droplet flow can be more broadly applied to liquid flows in general, with applications in improving insulation systems [29] and enhancing the understanding of co-flowing gas-liquid flows [30]. Compared to our previous work [5], there are several novelties. First, we found the available analytical solutions to different terms in our current theory of the slit. Notably, we used the analytical theory by Roscoe in 1949 [25] on flow passing a slit, which was the counterpart of the Sampson theory of a circular pore [31]. This is very significant in terms of fundamental transport theory in fluid mechanics. Second, from the application perspective, the current work is much more closely related to our recent work on the study of red blood cells passing through IESs in the spleen [2]. The new result in our current study, such as Equation (28), gives more precise relationships between transit times (total and for each stage) and slit dimensions than a circular pore theory. This will give more insights into explaining the dynamics of cells passing through slits observed in our experiments and simulations, because it explicitly gives relationships which cannot be obtained in numerical simulations. Third, for the surface tension terms, we incorporated the recent result from Darvishzadeh et al. [26] for applying the Young–Laplace equation to a rectangular opening, which is quite different from applying the Young–Laplace equation to a circular pore. Fourth, for the lubrication thickness, we utilized the result for a droplet inside a Hele–Shaw flow, which is different from a droplet in a circular tube. Finally, besides the parametric studies of transit times, we also showed detailed temporal evolution of the droplet of volumes in Figure 5, which was not carried out in the previous work.

Although we considered more realistic geometry in this study, there remain some limitations. While our expression evaluates v as a sphere, $v_{0}$ in the beginning of stage III is actually a hemispherical cap on the right side. As a result, the expression for $∆P_{mem}$ appears larger and our analytical model does not perform as well when the pressure drop approaches ${∆P}_{c}$. This effect can be seen as otherwise inexplicable sudden flow rate changes in Figure 5 not associated with the droplet initially or fully crossing a boundary. The sudden change in curvature calculation between stages appears to be a sharp change in flow rate between stages II and III for *V_II_* and between stages III and IV for *V_III_*. In addition, the calculation for critical pressure, although it would theoretically be found with the initial conditions of stage II, it is ultimately determined by the initial conditions in stage III due to assuming a spherically shaped droplet end. It will be more accurate to consider a spherical cup to evaluate the left and right spheres in this study; however, this will significantly increase the complexity of the analytical expressions. Because this study focused on total transit time, and the fact that the time where the right and left caps are not approximately spherical is relatively short, our model was sufficiently accurate.

## Data Availability

All data are included in the article.
